# Prevalence and factors associated with the use of long-acting reversible and permanent contraceptive methods among women who desire no more children in high fertility countries in sub-saharan Africa

**DOI:** 10.1186/s12889-022-14575-x

**Published:** 2022-11-21

**Authors:** Obasanjo Afolabi Bolarinwa, Agani Afaya, Kobi V. Ajayi, Abimbola Ojo, Oluwatobi Abel Alawode

**Affiliations:** 1 Department of Global Public Health, Canterbury Christ Church University, Canterbury, UK; 2 Discipline of Public Health Medicine, School of Nursing and Public Health, University of KwaZulu-Natal, Durban, South Africa; 3 College of Nursing, Yonsei University, 50‑1, Yonsei‑ro, Seodaemun‑gu, 03722 Seoul, South Korea; 4 Department of Nursing, School of Nursing and Midwifery, University of Health and Allied Sciences, Ho, Ghana; 5 Department of Health Behavior, School of Public Health, Texas A&M University, College Station, 77843 TX USA; 6 Department of Public Health, School of Health & Life Science, University of Teesside, Middlesbroug, UK; 7 Department of Sociology and Criminology & Law, University of Florida, 32611 Gainesville, FL USA

**Keywords:** Long-acting reversible contraceptives, Permanent contraceptives, Women who desire no more children, Sub-Saharan Africa, DHS

## Abstract

**Background:**

The proportion of women with family planning needs increased from 74 to 76% between 2000 and 2019, and this improvement has not transcended to a fertility rate stall or decrease in sub-Saharan Africa (SSA). In the face of a continuous increase in the fertility rate in SSA, population experts agreed that the efficient use of reliable family planning methods such as long-acting reversible and permanent contraceptive methods (LARC/PMs) could help reduce the high fertility rate and associated adverse reproductive health outcomes in the region. However, despite the effectiveness of LARC/PMs, its use remains elusive in SSA. Thus, this study examines the prevalence and factors associated with the use of LARC/PMs among women who desire no more children in high-fertility countries in SSA.

**Methods:**

Secondary datasets from the demographic health surveys conducted in 20 countries in SSA between 2010 and 2019 were included in the study. A total sample size of 46,290 sexually active women of reproductive age who desire no more children and who met the study inclusion criteria was pooled and analysed. Prevalence of LARC/PMs use was displayed using a graph whilst binary logistic regression was used to determine the associated factors, and results were presented as unadjusted odds ratio and adjusted odds ratio with a statistical significance of *p* < 0.05.

**Results:**

The prevalence of LARC/PMs use among women who desire no more children was 7.5%. Ranging from 20.9% in Senegal and as low as 0.4% in Congo. Women within the richest wealth index [aOR = 1.18, 95% CI = 1.03–1.36] and those exposed to mass media [aOR = 1.54, 95% CI = 1.41–1.68] had higher odds of LARC/PMs use among sexually active women of reproductive age who desire no more children compared to those within poorest wealth index and women with no mass media exposure.

**Conclusion:**

The study concluded that LARC/PMs use among sexually active women who desire no more children was very low, and women within the richest wealth index and those with mass media exposure were likely to use LARC/PMs. Interventions that will encourage using LARC/PMs should be prioritised to reduce fertility rates in SSA.

## Background

Even though the proportion of women worldwide whose family planning needs were satisfied slightly increased from 74 to 76% between 2000 and 2019, no noticeable decline was found among those with unmet family planning needs [1]. Access to family planning, including long-acting reversible and permanent contraceptive methods (LARC/PMs), are essential sexual and reproductive health commodities crucial to achieving the United Nations (UN) Sustainable Development Goals (SDG) 3 by 2030 because of their benefits in averting adverse maternal and neonatal outcomes [2–4].

LARC/PMs comprise of four contraceptive methods: Intra-uterine devices (IUDs), implants, tubal ligation, and vasectomy. Whereas IUDs and implants are long-acting reversible methods that can be removed depending on the type, tubal ligation and vasectomy are permanent methods [5, 6]. Together, these methods have clear advantages over short-acting and traditional methods of contraception because of their efficacy and effectiveness [7].

It is well documented that regional disparities in access to and use of contraceptives exist, with low contraceptive prevalence and high unmet family planning needs concentrated in sub-Sahara Africa (SSA) region [8]. For example, over 20% of the global unmet needs for family planning are concentrated in 15 countries in SSA [1]. According to Bradley et al., [9] unmet need for family planning can be defined as the percentage of all fecund married women or women living with a sexual partner and not using a method of contraception even though they do not want to get pregnant while satisfied demand family planning is regarded as women who are using contraception are considered to have a met need for family planning [10]. Interestingly, it is projected that contraceptive use prevalence will increase across regions of SSA by 2030; it will increase from 17 to 27% in West Africa, it is expected to rise from 40 to 55% in Eastern Africa, and 23 to 34% in Middle Africa [4]. Still, this projected increase would not suffice to reduce the projected levels of unmet family planning needs in the region [4, 11]. Also, the population growth in SSA is expected to increase the global unmet contraceptive needs by 2030 to 143 million, up from 142 million in 2015 [4].

Fertility rates are measures of understanding population and economic growth, which could have serious implications for sexual and reproductive health outcomes, particularly maternal and infant health. Using national survey data, researchers found that SSA had the overall highest fertility rate and age-specific fertility rates among 70 countries in Africa, Asia, and Latin America [11]. Correspondingly, according to the World Bank, 10 SSA countries alone account for the countries with fertility rates of 5.0, higher than global estimates of 2.4 children per woman and the average rate of 4.8 in SSA [12]. Because high fertility rates have a downstream effect on reproductive health, experts agree that access to family planning services and counselling in SSA holds promise to reduce the alarmingly high fertility rates in the region [11, 13], thereby improving overall maternal and infant health outcomes in the regions.

Considering the association between family planning use and high fertility rates [8, 14], it is imperative to examine its predictors among women who desire no more children in SSA countries with high fertility rates. Indeed, the use of LARC/PMs affords women the opportunity to make an informed decision to choose whether to limit or space childbearing [15, 16]. This is important because, in SSA, high fertility clusters cut across different spatially close countries, suggesting that focusing on country-specific family planning needs may not accurately reflect the disparities and variations in access to contraceptives in the region [11]. Yet previous studies on the predictors of LARC/PMs use have focused on specific countries [5, 17–19]. Furthermore, disparities in the use of LARC/PMs, despite their effectiveness in improving maternal and infant health, have been reported in the SSA [20, 21].

Because access to LARC/PMs remains elusive in contrast to other contraception methods in SSA [5, 22], this study adds to the literature by examining the predictors of LAPC/PMs use among women who desire no more children in high fertility countries to suggest targeted public health recommendations that are useful to these countries.

## Methods and materials

### Study design and data source

The latest Demographic Health Surveys (DHSs) from the countries included were utilised for the study. The DHS conducted in each country was a well-representative sample of the country using a cross-sectional survey design. Questions on socio-demographic characteristics and other sexual and reproductive health-related indicators, such as family planning use, intimate partner violence, abortion etc., were asked using a questionnaire among women of reproductive age between the age of 15–49 [23]. The survey design involves a two-stage sampling procedure, and the primary survey unit was made up of a sample drawn randomly from clusters in each country included in the study. The inclusion criteria for the study respondents include being a sexually active woman of reproductive age (15–49 years) and having no desire for more children, resulting in a total of 46,290 women in 20 high fertility countries in SSA, as shown in Table 1.

More information on the broad DHS sampling pretest and methodology has been published elsewhere [7, 24]. The survey is a recognised good source of the secondary dataset in SSA and has been utilised by various previous studies conducted in the region [7, 25–29]. We adopted the observational studies epidemiology guidelines in writing and reporting the findings from this study [30]. The datasets utilised in this study are available in the public domain and can be requested and downloaded upon approval from https://dhsprogram.com/data/available-datasets.cfm.

### Study variables

#### Outcome variable

The outcome variable for this study was LARC/PMs. DHS asked about the type of contraceptive women aged 15–49 use, and those who were using an intrauterine device (IUD), implant and tubal litigation were categorised as using LARC/PMs, and other types of contraceptive use were categorised as not using LARC/PMs. The use of LARC/PMs or not was restricted to sexually active women who desire no more children in high fertility countries in SSA, as it has been measured in similar studies [7, 21, 31].

#### Explanatory variables

Explanatory variables included in this study were selected based on the availability in the dataset, previous studies, and theoretical and practical significance [7, 32, 33]. These variables were the age of respondents, type of place of residence, household wealth index, household power relations, exposure to mass media, partner’s level of education, women’s level of education, age at first marriage and age at first birth.

The age of respondents were categorised as 15–24 years, 25–34 years, and 35 years and above. Types of places of residence were urban and rural areas. The DHS operationalised the household wealth index based on the items available to each household. This was grouped into the poorest, poorer, middle, richer, and richest using principal component analysis (PCA) [34]. Household power relations were grouped into women involved and women not involved. Women’s exposure to mass media was categorised into “yes” for those who had exposure to at least television, radio, or newspaper, while those without any were categorised as “no”. Partner’s and women’s level of education was grouped into none, primary, and secondary/higher. Age at first marriage was categorised as “<18 years” for those who married less than 18 years and “18 years and above” for women who married 18 years and above, while age at first birth was categorised as “ less than 20 years” for those who gave birth at age less than 20 years and “20 years and above” for those who gave birth at age 20 years and above [7, 32, 33].

### Statistical analyses

The study followed three steps in analysing the datasets included in this study using STATA 17.0 version. After appending all the DHS datasets with the available choice of variables in SSA. First, the prevalence of LARC/PMs use among sexually active women who desire no more children in SSA was generated and represented with a graph. Then, the included explanatory variables were cross-tabulated with LARC/PMs use. The results were presented in a frequency distribution, percentages, and chi-square (*X*^*2*^), including explanatory variables, results with *X*^*2*^ *p*-value less than 1 were included in the binary logistic regression. Two models were constructed using binary logistic regression analysis. “Model I” was the unadjusted model (cOR) known as bivariate logistic regression, while “model II” was the adjusted model (aOR) known as multivariable logistic regression, which accounts for the association of the explanatory variables included in the study. Binary logistics regression was employed in this study because the study’s outcome variable (LARC/PMs) was measured in a binary factor. The regression analysis results were presented in cOR and aOR with their corresponding confidence interval (CI), which signifies the precision and significance of the reported models. The survey sample weight was applied to control for under-sampling and non-responses. All analyses were performed with STATA 17.0 (StataCorp, College Station, TX, USA).

**Table 1:** Distribution of countries included in the study by year of survey, weighted sample size, and total fertility rate

**Survey Countries**	**Survey Year**	**Weighted Sample**	**Percentage**	**Total fertility rate**
**Central Africa**				
Angola	2016	2431	5.25	6.2
Congo DR	2014	2680	5.79	6.6
Republic of Congo	2012	1100	2.38	5.1
Cameroon	2019	1848	3.99	4.8
Chad	2015	1532	3.31	6.4
**West Africa**				
Burkina Faso	2010	3171	6.85	6.0
Benin	2018	2691	5.81	5.7
Cote D’Ivoire	2012	1277	2.76	5.0
Gambia	2019	1224	2.64	4.4
Guinea	2018	1347	2.91	4.8
Liberia	2019	1372	2.96	4.7
Mali	2018	1700	3.67	6.3
Nigeria	2018	6995	15.11	5.3
Niger	2012	816	1.76	7.7
Togo	2014	1990	4.30	4.8
Senegal	2018	1040	2.25	4.4
**East & Southern Africa**				
Burundi	2017	4362	9.42	5.5
Tanzania	2016	2100	4.54	5.2
Uganda	2016	3906	8.44	5.4
Zambia	2018	2708	5.85	4.7

The table included sexually active women in high fertility countries in SSA who do not want more children

## Results

**Fig. 1:**
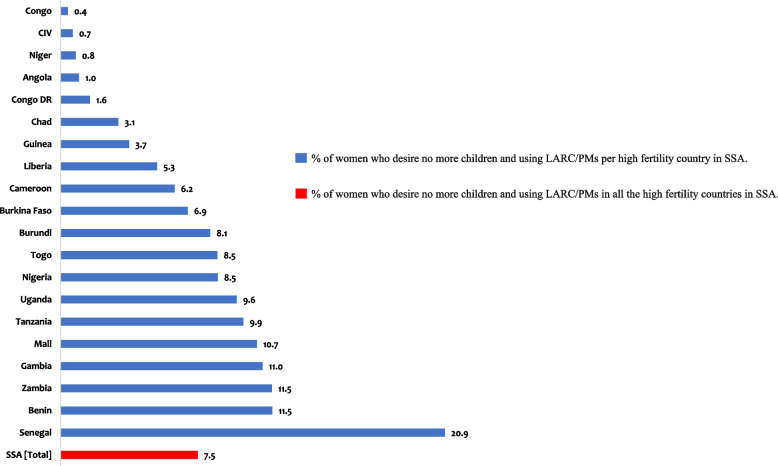
Prevalence of long-acting reversible and permanent contraceptive methods use among women who desire no more children in high fertility countries in SSA

Figure 1 shows the prevalence of LARC/PMs use among women who want no more children in SSA. The overall prevalence of LARC/PMs among women who want no more children in the 20 SSA countries considered in this study was 7.5%. For country-level prevalence, Senegal had the highest prevalence of LARC/PMs, with 20.9%, while the Republic of Congo (0.4%) had the lowest prevalence.

Table 2 shows the distribution of respondents’ characteristics. More than 70% of the respondents are 35 years or older, while 62% reportedly reside in rural areas. It can also be reported that 18% of the respondents are from the poorest households, while 21% are from the richest households. 64% of the women who desire no more children reported that they are not involved in decision-making in their households, the percentage exposed to any form of mass media is 67%, and 22% have completed secondary or higher education. More than half (51%) got married when they were older than 18 years of age, while 61% had their first birth at less than 20 years of age.

Considering the distribution of the use of LARC/PMs among women who desire no more children by explanatory variables., approximately 4% of this group of women aged 15–24 were using LARC/PMs, and 8% of those aged 25–34 and above 35 years were using LARC/PMs. The use of LARC/PMs among women who desire no more children in urban areas was 8.4% compared to 6.9% in rural areas. All the included explanatory variables in the study were significant at 0.001 except age at first birth, with a *p*-value greater than 0.05.

**Table 2:** Distribution of respondents by socio-demographic characteristics and use of long-acting reversible & permanent contraceptive methods [*n* = 46,290]

			**Long-acting reversible & permanent contraceptive use (%)**		
**Variables**	**Freq. [** ***n*** ** = 46,290]**	**Percent [%]**	**No**	**Yes**	***X***^**2 **^ ***p*** **-value**
**Age**					***p*< 0.001**
15–24	1,811	3.9	95.9	4.1	
25–34	11,903	25.7	92.2	7.8	
35+	32,576	70.4	92.5	7.5	
**Type of Place of Residence**					***p*< 0.001**
Urban	17,407	37.6	91.6	8.4	
Rural	28,883	62.4	93.1	6.9	
**Household Wealth Index**					***p*< 0.001**
poorest	8,385	18.1	93.7	6.3	
poorer	9,124	19.7	93.4	6.6	
middle	9,380	20.3	93.1	6.9	
richer	9,687	20.9	92.1	7.9	
richest	9,714	21.0	90.3	9.7	
**Maternal Position in Household decision making**					***p*< 0.001**
Woman Involved	16,503	35.7	93.6	6.4	
Woman Not Involved	29,787	64.3	91.9	8.1	
**Mass Media Exposure**					***p<*** **0.001**
No	15,095	32.6	94.8	5.2	
Yes	31,195	67.4	91.4	8.6	
**Partner’s Education**					***p*< 0.001**
No education	16,575	35.8	93.9	6.1	
Primary	13,476	29.1	91.8	8.2	
s/Higher	16,239	35.1	91.7	8.3	
**Woman’s Level of Education**					***p*< 0.001**
No education	20,945	45.3	93.9	6.1	
Primary	15,010	32.4	91.7	8.3	
s/Higher	10,335	22.3	90.9	9.1	
**Age at first marriage**					**0.003**
< 18	22,655	48.9	92.9	7.1	
> 18	23,635	51.1	92.2	7.8	
**Age at first birth**					**0.178**
Below 20	28,219	61.0	92.7	7.3	
Above 20	18,071	39.0	92.3	7.7	

*P-values obtained from Pearson chi-square test

Table 3 shows the logistic regression analyses of the factors associated with long-acting reversible and permanent contraceptive methods among women who desire no more children. Model 1 was crude, looking at the individual effects of these explanatory variables on long-acting reversible and permanent contraceptive method use among women who desire no more children in high fertility contexts of SSA.

The result of the adjusted model showed that compared to young women [15-24], women who desire no more children aged 25–34 [aOR = 1.85, 95% CI = 1.44–2.38] and older than 35 years [aOR = 1.84, 95% CI = 1.44–2.34] were more likely to use LARC/PMs. Women who desire no more children from the richest households were significantly more likely to use LARC/PMs compared to women from the poorest households [aOR = 1.18, 95% CI = 1.03–1.36]. Women who desire no more children who are exposed to mass media [aOR = 1.54, 95% CI = 1.41–1.68] had higher odds of using LARC/PMs than those not exposed. Women who desire no more children who have primary [aOR = 1.22, 95% CI = 1.11–1.35] and secondary/higher education [aOR = 1.23, 95% CI = 1.09–1.38] levels of education were found to have higher odds of using LARC/PMs compared to women with no formal education. Women who desire no more children whose partners have primary education are more likely to use LARC/PMs compared to those married to partners with no formal education [aOR = 1.14, 95% CI = 1.03–1.27].

**Table 3:** Bivariate and multivariable models showing the factors associated with the use of long-acting reversible and permanent contraceptive methods among women who desire no more children in 20 SSA countries

**Variables**	**Model I**			**Model II**		
	**cOR**	**95% CI**	***P*** **-Value**	**aOR**	**[95% CI]**	***P*** **-Value**
**Age**						
15–24	1			1		
25–34	2.03 ***	[1.53–2.70]	< 0.001	1.85 ***	[1.44–2.38]	< 0.001
35+	1.89 ***	[1.44–2.49]	< 0.001	1.84 ***	[1.44–2.34]	< 0.001
**Type of place of Residence**						
Urban	1					
Rural	0.79 ***	[0.71–0.87]	< 0.001	1.01 *	[0.93–1.11]	0.047
**Household Wealth Index**						
Poorest	1			1		
Poorer	1.04	[0.90–1.19]	0.592	0.97	[0.87–1.10]	0.672
Middle	1.16 *	[1.01–1.33]	0.041	0.96	[0.85–1.08]	0.453
Richer	1.35 ***	[1.17–1.56]	< 0.001	1.05	[0.93–1.18]	0.472
Richest	1.68 ***	[1.46–1.84]	< 0.001	1.18 **	[1.03–1.36]	0.015
**Household Power Relations**						
Woman Involved	1			1		
Woman Not Involved	1.30 ***	[1.18–1.42]	< 0.001	1.17 ***	[1.07–1.26]	< 0.001
**Exposure to Mass Media**						
No	1			1		
Yes	1.74 ***	[1.57–1.92]	< 0.001	1.54 ***	[1.41–1.68]	< 0.001
**Partner’s Level of Education**						
None	1			1		
Primary	1.35 ***	[1.21–1.51]	< 0.001	1.14 *	[1.03–1.27]	0.018
Secondary/Higher	1.44 ***	[1.29–1.61]	< 0.001	1.02	[0.91–1.14]	0.725
**Woman’s Level of Education**						
None	1			1		
Primary	1.41 ***	[1.28–1.56]	< 0.001	1.22 ***	[1.11–1.35]	< 0.001
s/Higher	1.69 ***	[1.50–1.90]	< 0.001	1.23 ***	[1.09–1.38]	0.001
**Age at first marriage**						
< 18 years	1			1		
> 18 years	1.14 ***	[1.05–1.24]	0.001	1.03	[0.94–1.13]	0.494
**Age at first birth**						
Less than 20 years	1			1		
Above 20	1.08	[1.00–1.18]	0.061	0.96	[0.87–1.05]	0.331

Model 1: Unadjusted model investigating the effect of each respondent’s characteristics on LARC/PMs use among women who desire no more children; Model 2: Adjusted model investigating the factors associated with LARC/PMs use among women who desire no more children. *95% CI* 95% Confidence Interval, *cOR* crude Odds ratio, *aOR* adjusted odds ratio

*p < 0.05

**p < 0.01

***p < 0.001

## Discussion

This study investigated the prevalence and factors associated with the use of LARC/PMs among women who desire no more children in high-fertility countries in SSA. The overall prevalence of LARC/PMs among women who desire no more children was 7.5%. Our study finding is similar to findings in Bangladesh (8%) [35] but lower than findings in Indonesia (28%) [36], India (43%) [35], Nepal (27%) [35] and other parts of Africa [37]. For country-level prevalence, Senegal had the highest prevalence of LARC/PMs, with 20.9%, while the Republic of Congo (0.4%) had the lowest prevalence. We observed varying levels of prevalence among the 20 SSA countries. Our findings resonate with previous studies that have established significant variation in the use of contraceptives in SSA [7, 38]. We may attribute these variations and low prevalence rates to geographical differences, access to LARC/PMs services, and country-specific programs targeting contraceptive use among women who desire no more children. Also, the varied prevalence of LARC/PMs use may be due to traditional, cultural, and religious prohibitions that have a strong connotation in women seeking contraceptive use in SSA [39]. The low prevalence rate of LARC/PMs use among women who desire no more children calls for action among sub-Saharan African nations. Evidence suggests that to improve the low prevalence rate of LARC/PMs use among women in low-resource settings, organized programs need to provide information about the benefits of LARC/PMs and access to those methods directly or through referrals [35]. It is evident that LARCs and PMs are typically unavailable in most rural or hard-to-reach areas due to lack of skilled providers, commodities, and equipment [40, 41]. Evidence demonstrates that mobile outreach services increase the use of contraceptives, especially in areas of low contraceptive prevalence, limited access to contraceptives, and also where geographic, social or economic barriers limit service uptake [42]. Therefore, well-designed mobile outreach services could help broaden the contraceptive method mix available to clients, including increasing access to LARC/PMs [40].

This study found that older women who desire no more children were more likely to utilize LARC/PMs. Our study finding is consistent with studies conducted in Nepal [43] and Indonesia [36], where younger women were less likely to use LARC/PMs compared to older women. Our study finding also resonates with other studies conducted within Africa [44, 45]. It is reported that younger women who desire no more children may be more concerned about the side effects of LARC/PMs use [39]. Sinai et al. [39] further noted that it’s possible that younger women with no intention of having any more children may have LARC/PMs prohibited by their partners or immediate/ external family members due to the strong cultural belief in maintaining the family lineage [39].

Consistent with our findings, studies conducted in Nepal [43] and Iran [46] revealed that women from the richest household are significantly more likely to use LARC/PMs than the poor. This finding may be due to financial barriers to accessing LARC/PMs in SSA, where most women are poor [47]. In developing countries, including most parts of SSA, some LARC/PMs are not easily accessible [47] except for implants which are readily found in community health centers. Most women will have to travel long distances to access LARC/PMs [47, 48]; therefore, the poor might not be able to afford the transportation cost to seek services. Our study contradicts findings from a multicountry study [35], where poor women in Bangladesh and India were more likely to use LARC/PMs. This divergence may be due to the different policy environments in these countries, where in some cases, there are supply-side and demand-side incentives with community mobilisation, that contributes to poor women’s uptake of these commodities. For example, in Bangladesh, the government has prioritized LARC/PM service delivery and has supported it with a large budget, including funds for client compensation and provider fees [49]. Also, in India, LARC/PMs are provided free of charge to the public [50] and have been a key part of the government promoting primary family planning methods [35].

A plethora of studies have demonstrated that exposure to mass media has the potential to influence an individual’s health behavior, especially with contraceptive usage [7, 38]. We found that women who were exposed to mass media were more likely to use LARC/PMs than those not exposed, which is consistent with a study conducted in India [51]. Evidence shows that access to different mass media outlets provides information that creates the awareness of women on family planning methods which in turn promotes its usage [51]. It was noted in India that television was the most effective means of disseminating family planning messages, followed by other mass media outlets [51]. The possible reason for this study finding is that in most recent times, family planning programs incorporate mass media campaigns that focus on delivering information about the various methods and educating women on their importance. As a result, women’s attitude toward contraceptive methods within SSA has improved [7, 20, 29, 38]. Therefore, we recommend using electronic and print media channels to disseminate LARC/PMs information among sub-Saharan African countries as this can increase LARC/PMs use among women who desire no more children. Usually, women in underserved communities often lack knowledge about LARC/PMs and have limited exposure to media and communication channels [40]. Therefore, sustained awareness-raising through mass media and community channels is critical to the success of the investments in family planning programs.

Our study findings suggest that women who desire no more children who have primary and secondary/higher education levels were more likely to use LARC/PMs than women without education. This study’s finding differs from a study conducted in Indonesia [36]. Our finding may be attributed to the fact that women with higher education may have more knowledge and information about LARC/PMs from several sources such as online and mass media, providers, or field workers [20, 52] and, as a result, will have more access to contraceptive services that match their reproductive goal. Also, educated women have more decision-making power regarding the family’s health, including contraception use [53]; however, the choice of discontinuation or none use in women with lower educational attainment could also be linked to possible side effects [54]. We also found that women whose partners had attained primary education were more likely to use LARC/PMs. Men exposed to various contraceptive methods through education have good knowledge and attitude towards using contraception for their partners. It is noted that men with higher education have good health-seeking behaviour and have the opportunity to discuss contraceptive use with health providers until they make the best decision for the appropriate contraceptive use that meets their goals [36]. Therefore, it is crucial to involve husbands or partners in family planning programs to provide full support for their partners in utilizing LARC/PMs if the client consents to involving her partner.

### Policy and public health implications

This study has several policies and public health implications for SSA. The study found a positive association between wealth and LARC/PMs use among women who desire no more children. This association is an issue of concern as this may signify inequity in access to LARC/PMs in many regions of SSA. Poor women were less likely to use LARC/PMs, possibly due to financial barriers which could be addressed by Governments or other relevant stakeholders providing financial subsidies on LARC/PMs services in SSA. Poor women may also have geographic barriers, requiring concerted efforts by multiple stakeholders to provide high-quality mobile outreach services to hard-to-reach areas. The study also found that mass media positively influenced the use of LARC/PMs. Therefore, concerted efforts by the public and private sectors are needed to provide messages focusing on the benefits of these methods through several media outlets to sensitize and create awareness about the availability of these services. Furthermore, we suggest that public health strategies and interventions on LARC/PMs should focus on young women and those with a low literacy level to improve the utilization of LARC/PMs services among this cadre of women. Lastly, this study has useful economic implications as comprehensive access to LARC/PMs in SSA may likely reduce the growing fertility rates in the region while also preventing adverse reproductive health outcomes.

### Strengths and limitations

The key strength of this study is the use of nationally representative data from the countries sampled for this study, making our findings generalizable to the population of women in these countries and SSA at large. Despite the strength, the study is fraught with some limitations. First, due to the study’s cross-sectional nature, we could not draw causal inferences between the outcome and explanatory variables. Considering the strong traditional and cultural inclination in most African settings in relation to contraceptive use, the study might be subjected to social desirability bias which might have influenced the outcome of LARC/PMs use among women who desire no more children. Despite the limitations, this study provides strong evidence of the prevalence and factors associated with the use of LARC/PMs among women who desire no more children in high-fertility countries in SSA.

## Conclusion

The study found a low and varied proportion of women who desire no more children utilizing LARC/PMs in 20 high fertility countries in SSA. Older age, household wealth, mass media exposure, higher education, and women whose husbands had primary education were predictors of LARC/PMs use. Therefore, Governments, policymakers, and relevant stakeholders could focus on these key predictors to implement public health policies, strategies, and intervention programs to improve LARC/PMs use among women who desire no more children in SSA.

## Data Availability

The datasets utilized in this study can be accessed at https://dhsprogram.com/data/available-datasets.cfm.
